# Poison With a Purpose: A Case Report on Arsenic Cardiotoxicity and Obesity

**DOI:** 10.7759/cureus.33185

**Published:** 2022-12-31

**Authors:** Emily A Nachtigal, Thanh Nga Doan

**Affiliations:** 1 Hematology and Oncology, Olive View University of California Los Angeles Medical Center, Los Angeles, USA

**Keywords:** obesity, tachycardia, cardiac toxicity, arsenic trioxide, acute promyelocytic leukemia

## Abstract

Acute promyelocytic leukemia (APL) is a form of leukemia in which there is an arrest of the maturation of the myeloid lineage at the promyelocyte stage. Although there is high early mortality due to coagulopathy, APL is now a curable disease with the use of arsenic trioxide (ATO) and all-trans-retinoic acid (ATRA). Arsenic is weight-based for the treatment of APL, and many toxicities are dose-dependent, although there are no guidelines regarding dosing adjustments for obese patients. We present a case of a 34-year-old male with obesity and APL who developed arsenic-induced QTc prolongation and symptomatic sinus tachycardia while receiving treatment. Further research is needed to guide appropriate dosing for obese patients to determine if ideal body weight dosing is able to provide similar cure rates with fewer adverse events.

## Introduction

Acute promyelocytic leukemia (APL) is a form of leukemia in which there is an arrest of the maturation of the myeloid lineage at the promyelocyte stage. In APL, there is a reciprocal translocation between chromosomes 15 and 17 that leads to a fusion of the promyelocytic leukemia (PML) gene with the retinoic acid receptor-alpha (RAR-alpha) gene that then codes for a protein that blocks cell differentiation, leading to APL [1]. High early mortality is seen due to hemorrhagic complications from coagulopathy, but APL is now very curable [2]. Arsenic trioxide (ATO) along with all-trans-retinoic acid (ATRA) with or without chemotherapy is the standard of care for the treatment of APL as it has been shown to induce APL cell differentiation and apoptosis with improved mortality when compared to chemotherapy alone [3]. We report a case of arsenic toxicity in an obese male (BMI 43.3 kg/m^2^) with weight-based dosing resulting in QTc prolongation and symptomatic sinus tachycardia.

## Case presentation

A 34-year-old male with obesity (BMI 43.3 kg/m^2^) and recent extensive occlusive superficial venous thrombosis of the right saphenous vein on rivaroxaban 10 mg presented to urgent care with hematuria and abdominal ecchymoses. Labs showed new anemia and thrombocytopenia. A review of the peripheral smear revealed blasts with Auer rods, and the patient was instructed to present to the emergency department for evaluation of acute leukemia. On arrival, he endorsed two to three weeks of easy bruising but denied any bleeding or "B" symptoms such as fever, night sweats, or weight loss. An exam revealed an obese male with diffuse ecchymoses on his abdomen and a palpable venous cord in his right lower extremity. Labs at the time of presentation can be seen in Table 1. Imaging revealed a nonocclusive deep venous thrombosis of the left common femoral and external iliac veins in addition to the known superficial thrombosis of the left greater saphenous vein. 

**Table 1:** Laboratory results at the presentation. PT: prothrombin time, INR: international normalized ratio, LDH: lactate dehydrogenase, PTT: partial thromboplastin time.

Lab	Value	Reference range
WBC (k/cumm)	7.4	4.5-10
Hgb (g/dL)	9	13.5-16.5
Plt (k/cumm)	61	160-360
PT (s)	23.2	11.8-14.4
INR	2.06	0.88-1.14
PTT (s)	37	23.6-36.2
Fibrinogen (mg/dL)	<60	215-450
D-dimer (mcg/mL)	>20	<0.49
LDH	888	98-192
Uric acid (mg/dL)	7.6	2.6-8

He was supported with transfusions and started on empiric ATRA due to concern for acute promyelocytic leukemia in the setting of blasts, Auer rods, and disseminated intravascular coagulation (DIC). A bone marrow biopsy was completed the following morning and confirmed the diagnosis of acute promyelocytic leukemia, with fluorescence in situ hybridization (FISH) demonstrating t(15;17) PML-RARα rearrangement. He was then started on induction therapy with ATRA and ATO per the Lo-Coco protocol [4]. The treatment was complicated by a pulmonary embolism (PE), leukocytosis requiring prednisone for differentiation prophylaxis, transaminitis, and neutropenia. Repeat bone marrow on day 22 of induction therapy revealed normal hematopoiesis without residual blasts by morphology and flow.

Due to the COVID-19 pandemic and patient distance from a healthcare facility, the patient was started on consolidation therapy with ATRA and ATO per the AML17 protocol, which is better suited for the outpatient setting [5]. Compared to Lo-Coco, the AML17 protocol gives higher doses of arsenic (0.3 mg/kg vs 0.15 mg/kg) over fewer visits, with overall similar arsenic dosage [6]. On week one, day four of consolidation therapy, the patient was found to have asymptomatic tachycardia with a heart rate of 130-140 s bpm with an EKG showing sinus tachycardia with prolonged QTc (491 ms), and treatment was held. His EKG prior to starting therapy showed borderline prolonged QTc (468 ms). He was not on any other QTc prolonging drugs. Electrolytes and thyroid-stimulating hormone (TSH) were within normal limits, a chest x-ray was unremarkable, and he was without any signs or symptoms of infection. Although he was on anticoagulation with rivaroxaban, a repeat chest computed tomography angiography (CTA) was completed to evaluate for a new PE and was negative. His heart rate remained elevated in the 140 s five days later, and he was ultimately admitted to the hospital for further monitoring and evaluation by cardiology. He was monitored on telemetry, which showed sinus tachycardia that improved with time. A transthoracic echocardiogram was completed and was unrevealing. He was temporarily on beta-blockers but was discharged home without the need for any rate control. As no other etiology was discovered and his heart rate improved with time, his sinus tachycardia with QTc prolongation was attributed to arsenic trioxide cardiotoxicity. He was then transitioned to consolidation therapy per the Lo-Coco protocol and completed therapy without QTc prolongation and with improvement in sinus tachycardia.

## Discussion

Arsenic is an environmental contaminant that has been used for medicinal purposes for over 2000 years; however, its use in APL was only discovered in the 1970s in China. Arsenic exerts its anti-tumor effects by inducing APL cell differentiation and apoptosis [1]. The FDA approved Trisenox, an injectable form of arsenic trioxide, in 2000 for the treatment of APL [7]. Despite its remarkable success in APL, arsenic is a known poison and carcinogen, and its use is limited by its toxicity [8]. Arsenic has known hepatotoxicity and cardiotoxicity and can less frequently cause dermatologic, neurologic, and gastrointestinal side effects [9,10].

Cardiovascular toxicities from arsenic include prolonged QTc, torsades de pointes, heart block, and even sudden cardiac death [11,12]. In a study evaluating the cardiotoxicity of arsenic in rats, the toxicity was dose-dependent and even seen at therapeutic levels [13]. Although the mechanism of action of cardiotoxicity from arsenic is not completely understood, a rat model proposed that the generation of free radicals causes structural changes due to edema from inflammatory cells [13]. Cardiotoxicity is also thought to result from the disruption of normal calcium and potassium homeostasis and mitochondrial dysfunction [8,14,15]. 

Tachycardia occurred in 55% of patients who received ATO at a dose of 0.15 mg/kg/d per package insert, making it an expected side effect [16]. Given that the AML-17 protocol administers arsenic at a higher dose (0.3 mg/kg/d), it is plausible that tachycardia might be more frequent or severe in that setting. QTc prolongation is seen in up to 11% of patients with APL undergoing first-line therapy with ATO/ATRA. Therefore, it is recommended to discontinue or at least minimize other QTc-prolonging medications [1]. Several medical organizations, as well as the package insert, provide guidelines for EKG monitoring and electrolyte replacement throughout treatment [2,16,17].

Obesity is a risk factor for APL, and our patient had a BMI of 43.3 kg/m^2^; he weighed 158 kg at diagnosis, and his ideal body weight was 79 kg [18]. This is relevant as ATO is typically dosed based on actual body weight, and toxicities from ATO occur at a higher frequency among obese patients [19]. In phase I and II study evaluating ATO dosing in patients with APL, three of 10 patients died of sudden cardiac death, with all of them being obese (>150% ideal body weight) [20]. As a result, some trials dose arsenic based on the ideal body weight for patients with a BMI >30 [19]. However, there are no organizational guidelines or randomized controlled trials to argue for dose modification of arsenic in obese patients with APL.

## Conclusions

There is little guidance on how to dose arsenic in obese patients, and given the high rates of complete remission and disease-free survival with treatment, underdosing could impact the chance for a cure. It is important to recognize the possibility of overdosing obese patients and to monitor closely for toxicities. Providers should consider using a regimen such as Lo-Coco with lower but more frequent dosing of arsenic for obese patients, as it was shown to minimize toxicity in our patients. Further research is needed to guide appropriate dosing for obese patients to determine if ideal body weight dosing provides similar cure rates with fewer adverse events.
